# Block‐wise Exploration of Molecular Descriptors with Multi‐block Orthogonal Component Analysis (MOCA)

**DOI:** 10.1002/minf.202100165

**Published:** 2021-12-08

**Authors:** Sebastian Schmidt, Michael Schindler, Lennart Eriksson

**Affiliations:** ^1^ Bayer AG, Crop Science Division, Environmental Safety Alfred-Nobel-Str. 50 40789 Monheim Germany; ^2^ Sartorius Stedim Data Analytics AB Östra Strandgatan 24 SE-903 33 Umeå Sweden

**Keywords:** Data analysis, Environmental chemistry, Molecular descriptors, Molecular modeling, Structure-activity relationships

## Abstract

Data tables for machine learning and structure‐activity relationship modelling (QSAR) are often naturally organized in blocks of data, where multiple molecular representations or sets of descriptors form the blocks. Multi‐block Orthogonal Component Analysis (MOCA), a new analytical tool, can be used to explore such data structures in a single model, identifying principal components that are unique to a single block or joint over multiple blocks. We applied MOCA to two sets of 550 and 300 molecules and up to 9213 molecular descriptors organized in 11 blocks. The MOCA models reveal relationships between the blocks and overarching trends across the whole dataset. Based on the MOCA joint components, we propose a quantitative metric for the redundancy of blocks, useful for *a priori* block‐wise feature selection or evaluation of new molecular representations. The second data set includes 7 ecotoxicological study endpoints for crop protection chemicals, for which we (re‐)discovered some general trends and linked them to molecular properties. Using a single MOCA model we estimated the predictive potential of each block and the model‐ability of the target block.

## Graphical abstract

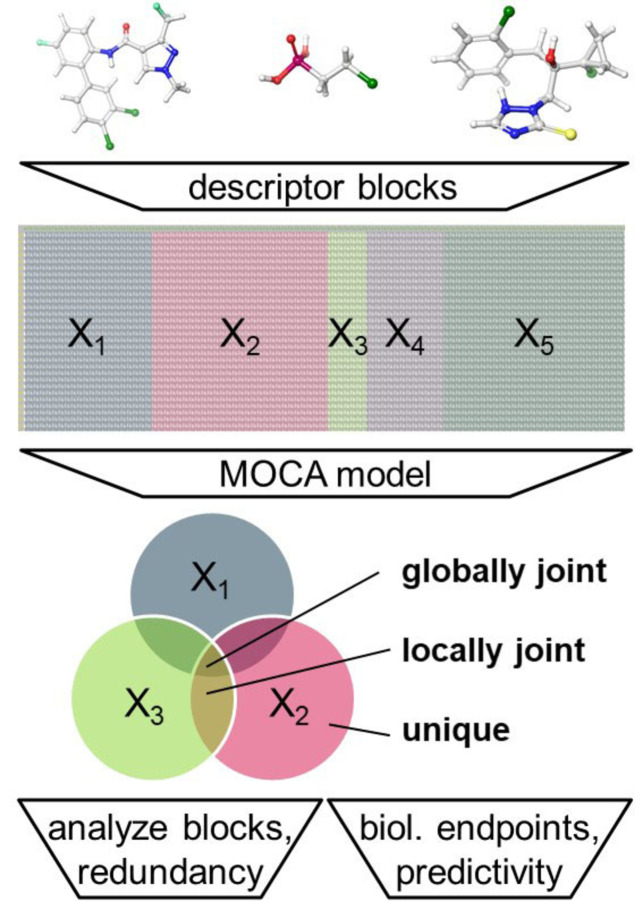

## 1 Introduction

Quantitative structure‐activity relationship (QSAR) modelling is the task of predicting a molecule's activity, e. g. a certain chemical reactivity or biological activity, from its molecular structure. The latter is usually represented by molecular descriptors, which quantify certain features of the molecular structure. A large variety of descriptors have been developed over the past decades, ranging from topology, shape, atomic properties, pharmacophores or toxophores, to fingerprints or fragment counts.[^1^, ^2^] With the recent technological advances of deep neural networks and natural language processing abstract machine‐learned representations or characters of structure notations like SMILES codes have become widely used.[^3^, ^4^] The choice of the molecular representation is a critical step in QSAR modelling and remains an expert decision.^[2]^ Oftentimes, this selection is based on the ease of availability of the tools to the model developer or the model performance on a predictive task.[^5^, ^6^, ^7^] The brute force approach of combining all available representations is not feasible due to computational constraints and the risk of spurious chance correlations with increasing numbers of input features. Therefore, we seek methods to inform the expert decision *a priori*, i. e. before any predictive models are built, e. g. by analysing the differences and similarities of chemical representations across a representative chemical space. This can also help to investigate the many claims in the literature that one chemical representation is superior, by working out their novel and unique information content in a systematic way, complementary to predictive performance.

To analyze large data tables with multiple blocks of descriptors efficiently, multivariate data analysis (MVDA) by projections methods is useful.

Broadly speaking there are four basic data analytical problem types to which these multivariate tools can be applied. (1) In the early stages of a project one often requires a simple overview of the information in a data table. Such an overview can be obtained with principal components analysis (PCA). (2) Another common data analytical problem is the two‐block (X/Y) regression problem, which can be addressed using partial least squares (PLS) or an extension called orthogonal PLS (OPLS). (3) A consequence of the results from an initial data analysis can be that sub‐groups or clusters of compounds are found that warrant further study in terms of local class models. To accomplish this objective, variants of PCA, PLS, or OPLS are often used. PCA, PLS and OPLS are explained in more detail in Section 2.3. (4) The fourth data analytical objective occurs when the data at hand are organized as multiple blocks. Multivariate models involving large numbers of variables are often difficult to interpret, because plots become cluttered and difficult to overview. There may then be a temptation to eliminate variables which runs the risk of losing important information and may make the modelling efforts more difficult to interpret and less robust. Usually, a better alternative is to partition the data into blocks of logically related variables and apply hierarchical[^8^, ^9^, ^10^, ^11^] or multi‐block data analysis,[^12^, ^13^, ^14^] or PLS path modelling if data are available as logical pathways of dependencies or time orders.^[15]^

The early cases of multiblock analysis[^8^, ^9^, ^10^, ^11^, ^12^, ^13^, ^14^, ^15^] had a strong focus on concatenating, summarizing and quantifying correlations among all data blocks, and sometimes to predict one block from one or more of the other blocks. In more recent years, however, the focus has shifted towards data integration in the sense of separating information that is common to many blocks from information that is distinctive to a certain block of data, and expressing such information in different model compartments, i. e. different model components. These attempts of separating joint and unique variabilities from each other stretches from approaches involving two blocks[^16^, ^17^] over to many‐block methods, such as JIVE,^[18]^ DISCO,^[19]^ OnPLS,[^20^, ^21^] and JUMBA/MOCA.^[22]^

Datasets with multiple blocks of variables may also be analyzed by hierarchical extensions of PCA, PLS, and OPLS.^[23]^ At the base level, detailed information is extracted for each block using block‐specific models. Subsequently, the scores formed at the base level are concatenated at the top level and analyzed by an overall model summarizing the information encoded by all base level score vectors. This top‐level model provides a condensed overview of the information found in all data blocks. One limitation of the hierarchical approach, however, is that it does not lend itself to readily labelling and quantifying the proportion of joint and unique variabilities in the data.

In this paper, we use a new embodiment of the OnPLS method denoted MOCA, short for Multiblock Orthogonal Component Analysis, as a navigation tool to block‐wise investigate a selection of molecular descriptor packages, which are frequently used as chemical representations in QSAR modelling works. We selected the MOCA method as it ties in nicely to the modelling spirit of the other multivariate methods employed in this work. Our selection of descriptors is exemplary and can be extended to other packages or chemical representations. We hope to encourage others to follow us along the path of better informing the selection of input features based on other criteria than predictive performance, and maybe spark ideas for the design of better chemical representations.

## 2 Computational Methods

### 2.1 Data Sets

We compiled two data sets, referred to as ChemGPS and Pesticides. The ChemGPS data set is an updated version of the published ChemGPS[^23^, ^24^] set of reference compounds, which was designed to navigate the organic molecules chemical space to support computational molecular design and other tasks. It is a principal component analysis (PCA) based system, where the 9 principal components represent physical‐chemical properties like molecular size, lipophilicity, and flexibility. The molecules were selected in a way to span a broad and diverse chemical space of organic molecules, yielding stable and interpretable principal components. We consider the ChemGPS reference set a robust and representative starting point for our analysis. Compared to the original extended set of 554 molecules, however, we removed 4 molecules (#484, #327, #205, #456), which became duplicates after our pre‐processing of the structures (see below) and corrected the structure of Indolapril (#6) for a hypervalent nitrogen atom. Furthermore, we used our own selection of molecular descriptors as X variables, as these are the subject of our analysis. In total, this set contains 550 molecules and 9213 molecular descriptor variables partitioned into 11 blocks representing the software packages for descriptor calculation.

The Pesticides data set was extracted from the Pesticide Properties Data Base (PPDB, as of 08.09.2018),^[25]^ which is a publicly available data source for properties of active ingredients of crop protection products and their metabolites. The properties range from physico‐chemical to environmental fate to toxicological properties and general business relevant information. We were particularly interested in the ecotoxicological properties, which are of high interest for the development and risk assessments of crop protection products. To avoid too sparse a data matrix, we restricted the selection to 7 ecotoxicological endpoints and 300 compounds with the highest number of known endpoint values. Only values without prefix (i. e. “>” or “<”) and labelled with high reliability (PPDB quality parameter 4 and 5) were selected. The selected endpoints are summarized in Table 1. They represent concentrations or dose levels at which an effect was observed for 50 % of the tested individuals. The only exception is the bioconcentration factor, which describes the partitioning of the substance from water into fish. More information on the endpoints is available on the PPDB website.

**Table 1:** Experimental data for 7 ecotoxicological endpoints in the Pesticides data set (log‐transformed molar units).

endpoint	min	max	mean	missing values
Algae, EC50 growth after 72 h	−0.26	6.37	2.60	16.7 %
Aquatic plants (*Lemna gibba* and *Lemna minor*), EC50 after 7 days	−2.02	6.22	2.79	52.0 %
*Daphnia magna* acute, EC50 after 48 h	−2.43	7.39	2.47	10.0 %
Fish acute, LC50 after 96 h	−2.46	7.11	2.37	10.3 %
Rat acute oral, LD50	−1.87	3.34	−0.19	36.3 %
Birds acute, LD50	−2.10	3.31	−0.04	52.0 %
Bioconcentration factor (fish)	−1.87	3.51	0.45	35.3 %

All endpoints were converted to negative logarithmic molar quantities (‐log X), which is a commonly applied transformation in many structure‐activity‐relationship models, except for bioconcentration factor which was only log transformed (log BCF). In addition to this biological data, the same molecular descriptors were used as for the ChemGPS data set.

### 2.2 Molecular Descriptors

Molecular structures were standardized with RDKit^[26]^ to canonical SMILES, including neutralization and removal of ambiguous stereo information. Inorganic compounds and compounds containing heavy metals like Pb, Hg, Sn, or Cu were filtered out. Salts and solvent molecules were stripped off. Compounds were grouped neglecting stereo information, i. e. the mean of the log‐transformed endpoints from all stereo isomers was taken.

We calculated molecular descriptors, Morgan fingerprints (1024 bits, radius 2), ECFP4 fingerprints (1024 bits, diameter 4), and MACCS keys based on the pre‐processed SMILES codes with the CDK (v1.5)^[27]^ and RDKit (version 3.8.0)^[26]^ implementations in the KNIME Analytics Platform.^[28]^ Unity fingerprints were calculated with Sybyl.^[29]^ 3D molecular structures were optimized with density functional theory (Turbomole 7.0.1,^[30]^ BP86 functional, def2‐TZVP basis set, COSMO‐RS continuum model[^31^, ^32^] for solvent water). The latter was also used to compute several quantum‐chemical descriptors for the predominant protonation state at neutral pH. The 3D structures of the neutral molecules were used to calculate descriptors with the Dragon 7.0^[33]^ and PaDEL^[34]^ software packages. The Dragon software was recently superseded by its successor software alvaDesc,^[35]^ which can calculate 35 descriptors in addition to the ones included in Dragon. We included these additional descriptors as the “alva_add” block. Additionally, we calculated the continuous and data‐driven molecular descriptors (cddd) recently published by Winter et al.,^[4]^ which are based on the embedding of a deep learning translation model for molecular structures.

### 2.3 Data Analytical Methods

All results reported in this contribution were obtained using the SIMCA software,^[36]^ version 16, and using default procedures for data pre‐treatment, i. e., all variables were mean‐centered and scaled to unit variance prior to model calculations. SIMCA uses the NIPALS algorithm^[36]^ which means automatic handling of missing values, such that any missing value has zero residual and thus no leverage on the estimated model parameters. R2 values denote the fraction of explained variance.

#### Principal Component Analysis (PCA)

Principal component analysis is the mother method for multivariate data analysis by projection methodology.[^37^, ^38^, ^39^] PCA works with a single data table, **X**, with *N* rows (observations) and *K* columns (variables). The objective of PCA is to summarize the information content in a data table X. Such an overview model may reveal groups, clusters, trends, and deviators among the observations, as well as giving information about the correlation structure among the variables.

From a geometrical perspective, PCA can be understood as finding lines, planes and hyperplanes in the *K*‐dimensional variable space that approximate the observation data points as well as possible in the least squares sense. From a statistical perspective, by using PCA, a data matrix **X** is decomposed as

$$X=1{}^{*}{\bar{x}}^{'}+T{}^{*}P{}^{'}+E$$

The first term above, 1* ${\bar{x}}^{'}$ , corresponds to the variable averages and originates from the pre‐processing step of mean‐centering. The second term, the matrix product T*P′, expresses the systematic information in the data, and the third term, the residual matrix **E**, contains the unmodelled variation. The principal component scores of the first, second, third, …, components (**t_1_**, **t_2_**, **t_3_**, …) are columns of the score matrix **T**. These scores may be understood as new variables which summarize the original ones. To interpret the scores, one uses the loadings. The loadings of the first, second, third, …, components (**p_1_** ′, **p_2_** ′, **p_3_** ′, …) form the rows of the loading matrix **P**′. The loadings inform about how the original variables are linearly combined to form the scores, and thereby they account for the direction of the PC (hyper‐) plane with respect to the original **X**‐variables.

#### Partial Least Squares Projections to Latent Structures (PLS)

Partial least squares projections to latent structures, PLS, is a regression extension of PCA.[^40^, ^41^] The objective of PLS is to analyze two blocks of data, often denoted **X** (“predictors”) and **Y** (“responses”) and use the easy‐to‐get‐hold‐of data (**X**) to predict the difficult‐to‐get‐hold‐of data (**Y**). It should be noted that in many cases the Y‐data is not a matrix of response variables, but rather a single **y**‐variable.

PLS modeling of the relationship between two blocks of data can be described in different ways. One way to see PLS is that it computes two “PCA‐like” models, one for **X** and one for **Y**, and simultaneously aligns these models. The objectives are (a) to model **X** and **Y**, and (b) to predict **Y** from **X**, according to:

$$X=1{\bar{x}}^{'}+\mathrm{TP}{}^{'}+E$$

$$Y=1{\bar{y}}^{'}+\mathrm{UC}{}^{'}+F\left(=1{\bar{y}}^{'}+\mathrm{TC}{}^{'}+G,\;\mathrm{due}\;\mathrm{to}\;\mathrm{inner}\;\mathrm{relation}\right)$$

In the two expressions above, the first terms, **1** ${\bar{x}}^{'}$ and **1** ${\bar{y}}^{'}$ , represent the variable averages and originate from the pre‐processing step. The score matrices **T** and **U** contain the observation related information, and the **X**‐loading matrix **P′** and the **Y**‐loading matrix **C′** contain the variable related information. The variation in the data that was unmodeled form the **E** and **F** residual matrices.

#### Orthogonal Partial Least Squares (OPLS®)

Orthogonal PLS (OPLS®) is a modification of PLS allowing simplified model interpretability.[^42^, ^43^, ^44^] This is accomplished through the ability of OPLS to divide the systematic variation in the **X**‐block into two parts, one part which expresses the predictive correlations between **X** and **Y** and another part which expresses the variation in **X** that is not related (orthogonal) to **Y**. OPLS components that are correlated to **Y** are here called *predictive* whereas components that are uncorrelated to **Y** are called *orthogonal*. In case the OPLS model only covers a single **y**‐variable, by theory, there can then solely be one predictive component, but there can be any number of orthogonal components. This is why single‐**Y** OPLS models are so useful as there is only one predictive component to consider.

In the case of a single **y**‐variable, we can write the **X**‐part of the OPLS model as

$$X=1{\bar{x}}^{'}+t{}_{p}p{}_{p}{}^{'}+T{}_{o}P{}_{o}{}^{'}+E$$

and the OPLS model prediction of **y** as

$$y={\bar{y}}^{'}+t{}_{p}q{}_{p}{}^{'}+f$$

The OPLS model for multiple **Y**‐variables involves more elaborate matrix expressions, which are outside the scope of this article.

#### Multiblock Orthogonal Component Analysis (MOCA)

Multiblock Orthogonal Component Analysis (MOCA) is a new tool aimed at disentangling the information in complex multi‐block data analytics problems. It is available in the software SIMCA® version 16^[36]^ and is a specific embodiment of a data analytical procedure known as OnPLS in the scientific literature.[^20^, ^21^] Embodiments sometimes differ in algorithm tweaks, thresholds, or default values for adjustable parameters. Hence, by using the term MOCA (and not OnPLS or JUMBA), it is clear that our calculations have taken place in SIMCA®.^[36]^

Thus, MOCA is a result of long‐term method evolution among the family of orthogonal PLS methods. OPLS and O2PLS address the two‐block problem, O3PLS three data blocks and OnPLS ‘n’ blocks. The common denominator among these orthogonal methods is their ability to split information structures residing in data into correlated (joint) and uncorrelated (unique) sources of variabilities. This property makes the OnPLS embodiment MOCA particularly attractive to us.

**Figure 1:**
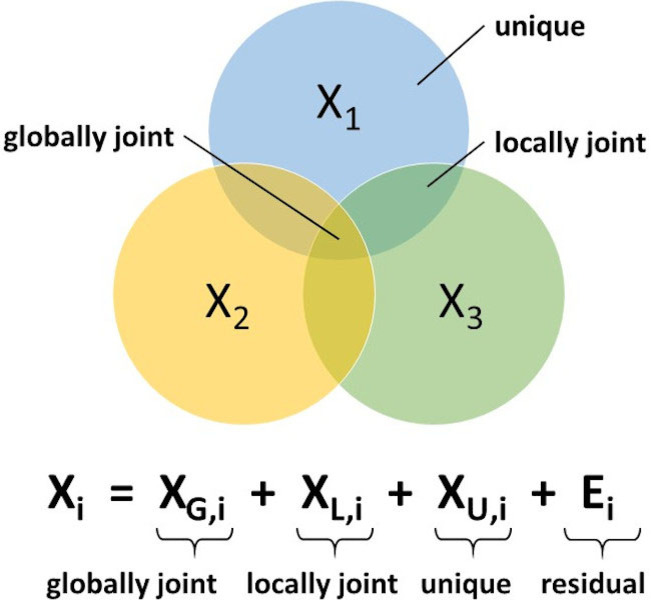
Venn diagram showing the data decomposition principle of the MOCA model using a fictitious three‐block data analytical problem. The MOCA model will have three model compartments. One type of components will express globally joint information found in all data blocks being analysed. A second type of components will represent locally joint information found in a subset of the data blocks. And a third type of components will capture unique information found only in one, single data block. The residual part corresponds to the unmodelled data structure of each data block, once the joint and unique variabilities have been accounted for.

Regardless of the number of data blocks covered by the current model, MOCA will extract two sets of components; joint and unique components.^[22]^ A schematic illustration of the MOCA model in the case of three data‐blocks is shown in Figure 1. More specifically, for every data block, X_i_, the variation is decomposed as globally joint information, locally joint information, and unique information, as shown in Figure 1.

- Globally joint information is systematic structure found in all data blocks being analyzed;
- Locally joint information is systematic structure found in a subset of the data blocks; and
- Unique information is additional systematic structure found only in one, single data block.

Briefly, the MOCA algorithm comprises five major steps:

1. The first step is to compute pairwise OPLS models of all included blocks to determine a suitable number of joint components for each pair of blocks. This implies the algorithm treats each data block according to equal weight, independent of their size.
2. In the second step, all OPLS components from step one are combined into one set for each individual data block. From each such set, PCA is then used to extract only the relevant number of components for each data block. These “compressed” components represent the joint information in each data block.
3. In the third step, using the new “compressed” components from step two, the joint information in each block can be recreated by multiplying the scores and loadings to recreate the block data. The difference between the recreated joint block matrices and the corresponding original block matrices is a set of “residual” blocks corresponding to the unique block information.
4. In the fourth step PCA is used to extract the unique components from the “residual” blocks remaining after the third step.
5. In the fifth step the joint information from step three is re‐assessed and divided into globally and locally joint components. This step involves a series of PCA and OPLS models. In this process, a specific final loading vector is evaluated to determine if the component under consideration is a globally or locally joint component.

The MOCA algorithm contains a strictness parameter which is linked to the final loading vector in Step 5 and influences the proportion of the data structure which will be regarded as joint information. It ranges from −1 to 0 and the closer to 0 the stricter the solution. In the strictest scenario, where high demands are placed on what is labelled as joint information, the individual block joint components of the overall joint components must be very similar. In a less strict scenario, where the goal might be to extract as much systematic information as possible, a less stringent view on what information is connected between different data blocks is applied.

Visualization of MOCA model parameters is similar to visualization of PCA/PLS/OPLS model parameters. This means plots of scores and loadings are frequently used for model interpretation. However, compared with ordinary PCA/PLS/OPLS score plots, the MOCA score plot of joint components offers a diagnostic advantage. For each joint component (global or local) it is possible to plot either a score vector pertaining to an individual data block, or the average joint score vector across all blocks. For the average joint score vectors, SIMCA sizes the points proportional to the block differences. A small point in the score plot indicates an observation (here: compound) for which the individual block score vectors are very similar to the average score vector. Conversely, a large point highlights larger differences. The size of the plotted point thus indicates how much joint information is preserved among the modelled data blocks for that particular compound, i. e., the smaller the point the higher degree of information overlap.

In terms of computation time MOCA is more demanding than PCA but not prohibitively more. Due to the initial pairwise modelling in step 1 the number of included blocks is a main driver of the computation time. The number of variables in each block usually has a much less decisive impact. Building the largest model with 550 molecules and 9213 descriptor variables in 11 blocks did not take longer than 30 minutes on a standard business laptop.

## 3 Results and Discussion

### 3.1 Assessing the Influence of the Strictness Parameter in MOCA Models

To investigate the influence of the strictness parameter additional calculations were done on the ChemGPS data set. Initially, a series of six 11‐block MOCA models was created in which the parameter adopted the values −0.01, −0.03, −0.05, −0.10, −0.20 and −0.50. For each of the six conditions the fraction of joint variation (R2Xj) was registered. Furthermore, to introduce some additional information possibly “disturbing” the system we also invoked a twelfth block of descriptor variables from an in‐house package. Using the 12‐block setup an additional series of six MOCA models were computed in which the strictness parameter was anew varied as −0.01, −0.03, −0.05, −0.10, −0.20 and −0.50. Again, the R2Xj fraction of joint variation was registered.

In the next stage, a data table (see Table 2) with the resulting R2Xj values was created in which each row was one condition and each column an R2Xj value for a particular block of descriptor variables. This means the data table had 12 rows (six settings of the strictness parameter times the two MOCA setups of 11‐blocks and 12‐blocks) and 11 columns (R2Xj‐values for the 11 descriptor blocks of interest).

**Table 2:** Fraction of joint variation (R2Xj) obtained when analysing the influence of the strictness parameter.

Nr.	Blocks	Strictness	RDKit	CDK	cddd	QM	Dragon	PaDEL	alva_add	ECFP4	unity	MACCS	Morgan
1	11	−0.01	0.394	0.531	0.331	0.256	0.592	0.560	0	0.121	0.239	0.179	0.122
2	11	−0.03	0.634	0.753	0.416	0.484	0.630	0.571	0.608	0.188	0.309	0.467	0.187
3	11	−0.05	0.725	0.847	0.435	0.843	0.634	0.586	0.667	0.182	0.302	0.525	0.187
4	11	−0.1	0.767	0.816	0.426	0.758	0.634	0.586	0.761	0.177	0.317	0.585	0.182
5	11	−0.2	0.799	0.896	0.435	0.743	0.634	0.583	0.836	0.188	0.323	0.605	0.192
6	11	−0.5	0.865	0.931	0.452	0.944	0.634	0.592	0.929	0.188	0.343	0.631	0.186
7	12	−0.01	0.673	0.684	0.358	0.298	0.619	0.565	0	0.153	0.258	0.202	0.153
8	12	−0.03	0.632	0.779	0.419	0.578	0.633	0.582	0.641	0.172	0.327	0.499	0.183
9	12	−0.05	0.727	0.779	0.418	0.645	0.625	0.580	0.558	0.177	0.300	0.539	0.177
10	12	−0.1	0.786	0.780	0.436	0.667	0.624	0.579	0.744	0.166	0.316	0.581	0.165
11	12	−0.2	0.800	0.868	0.435	0.895	0.620	0.579	0.858	0.170	0.329	0.618	0.169
12	12	−0.5	0.865	0.926	0.454	0.945	0.638	0.594	0.929	0.188	0.344	0.642	0.187

PCA was then applied to the data table of R2Xj‐values and of particular interest was the score plot. Prior to the PCA calculations the data table of R2Xj values was only mean‐centered (but not scaled to unit variance). Mean‐centering is the usual procedure when the data have a similar numerical range – 0‐1 in this case – and arise by the same underlying mechanism.

**Figure 2:**
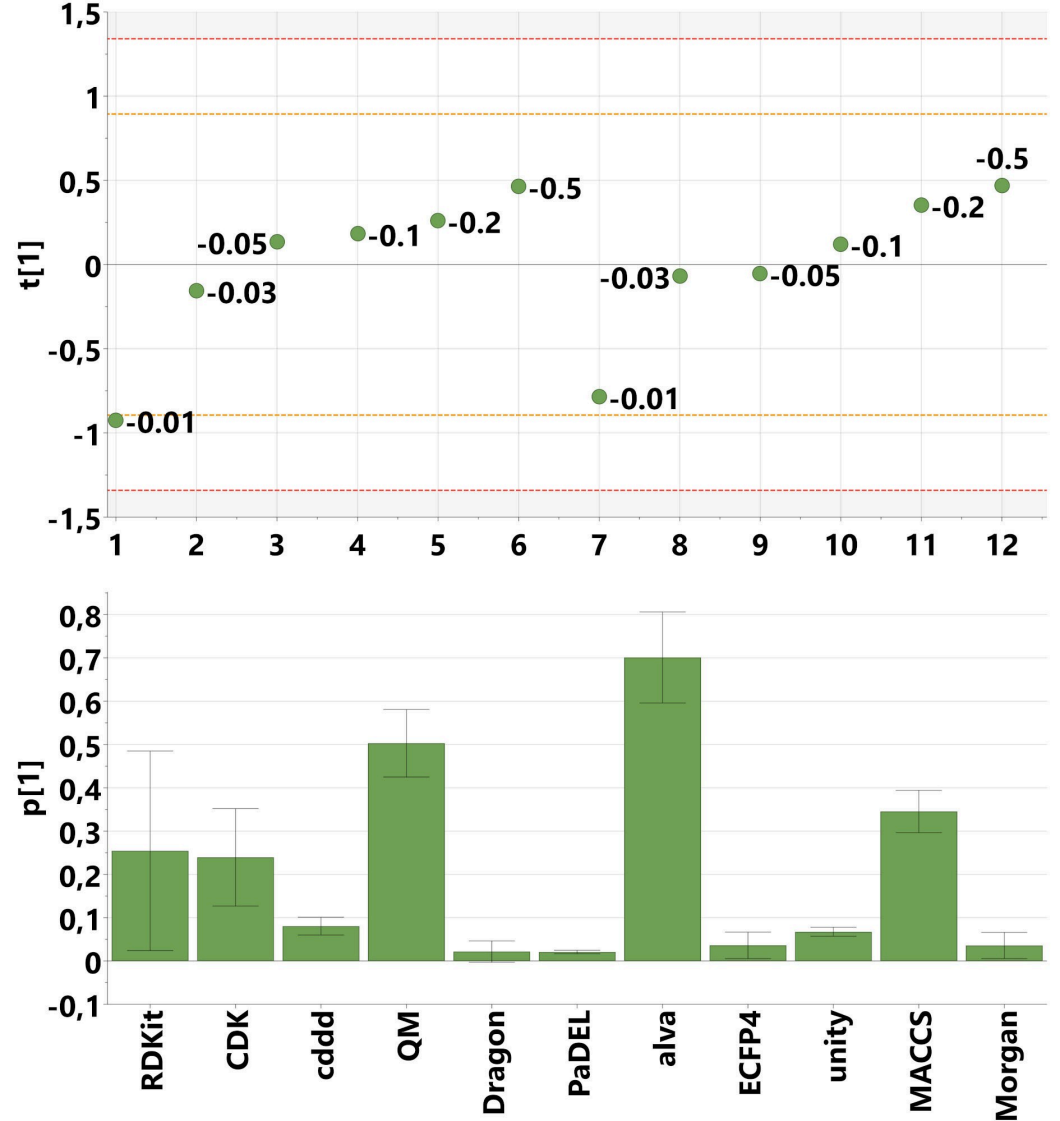
Results from assessing the influence of the strictness parameter. Top: Score plot. Model numbering is in accordance with Table 2. The numerical label is the setting used for the strictness parameter. Yellow and red horizontal lines represent 2 and 3 standard deviation limits. Bottom: Loading plot. Each column is the loading value of the descriptor block, the error bars represent 95 % confidence intervals as estimated by jack‐knifing.

The PCA model obtained had one strong and significant principal component accounting for 94 % of the variance in the data table of R2Xj‐values. Its score and loading plots are seen in Figure 2. The score plot shows two consistent, repeating trends of the numeric series 1‐6 (the six 11‐block MOCA models) and 7–12 (the six 12‐block MOCA models), indicating that addition of the 12^th^ block does not perturb the model qualitatively. In both numeric series cases, the model with the strictest solution (1 and 7) are somewhat remotely positioned from the rest of the models using the same set‐up. This suggests rather sharp changes in R2Xj‐values profiles (joint variabilities) when moving from the strictest solution given by the strictness −0.01 to the software default of −0.03. As opposed to this, however, models 2‐6 and 8‐12 display a trajectory indicating a rather stable continuum in how the R2Xj‐values (joint variabilities) change depending on the setting of the strictness parameter. This finding suggests that, as long as the strictest solution is avoided, the chosen setting of the strictness parameter will not fundamentally change the structure of the information overlap of the different block of descriptors. What will change is the proportion of descriptor variation diagnosed as joint and overlapping information.

Furthermore, it is of interest to interpret the loading vector of the PCA model (Figure 2, bottom). With the help of the loading vector we can sub‐group the different descriptor packages with respect to how sensitive or insensitive they are due to altering the setting of the strictness parameter. The larger the loading value the more the fraction of joint variation is growing when the strictness is relaxed. This means that the smallest descriptor blocks alva_add and QM and their joint variabilities are particularly sensitive to the choice of the strictness parameter, which we attribute to their small size. In the strictest setting the alva_add block did not contribute any joint variance. Conversely, for the large descriptor blocks Dragon, PaDEL, ECFP4 and Morgan comparatively stable fractions of block‐overlapping information is detected, i. e., the R2Xj values hardly change when modifying the strictness parameter.

Our conclusion is that the default setting of this parameter (−0,03) is sensible and provides faithful results. This default value is used in all MOCA models quoted in this work if not stated otherwise.

### 3.2 Analysis of Descriptor Blocks on a Reference Set of Molecules (ChemGPS Data Set)

**Figure 3:**
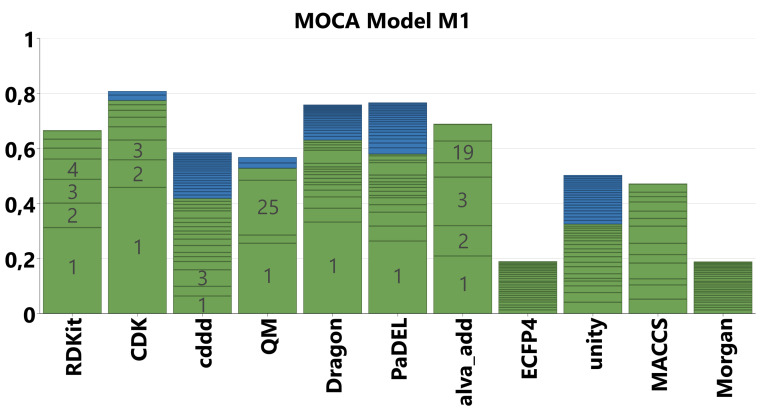
Overview of explained variance (R2) for the MOCA model M1 on the ChemGPS data set with 11 descriptor blocks. Segmentation reflects explained variance per joint (green) and unique (blue) component. Numbers of the major components are displayed in grey.

We started our analysis on the established ChemGPS^[24]^ set with the goal to understand the interrelationship between the various descriptor blocks and eventually to reduce the number of descriptor packages required for predictive modelling. The first MOCA model (M1) contains a total of 9213 molecular descriptors partitioned into 11 blocks representing the software packages for descriptor calculation. Figure 3 shows an overview of explained variance (R2) covered by the globally or locally joint components (green) and the unique components (blue) for each block.

Figure 3 depicts several characteristics of the descriptor blocks. The conventional descriptor types (RDKit, CDK, Dragon, PaDEL, alva_add) show very large fractions related to a single joint component, which represents molecular size (see below). On the other hand, the fingerprints (ECFP4, Morgan, unity, MACCS) as well as cddd show very small explained variance per component, which is due to their design to cover large chemical spaces using a limited number of (orthogonal) dimensions. We therefore decided to analyze the fingerprints in a separate model (see Supporting Information for details). Except the ECFP4 and Morgan blocks, which are very similar by design, i. e., both using the Morgan algorithm,[^45^, ^46^] all these blocks contribute unique information in terms of many fine‐grained (i. e., low R2) components. This nature of the fingerprints renders them more useful for large data sets (thousands of molecules).

**Figure 4:**
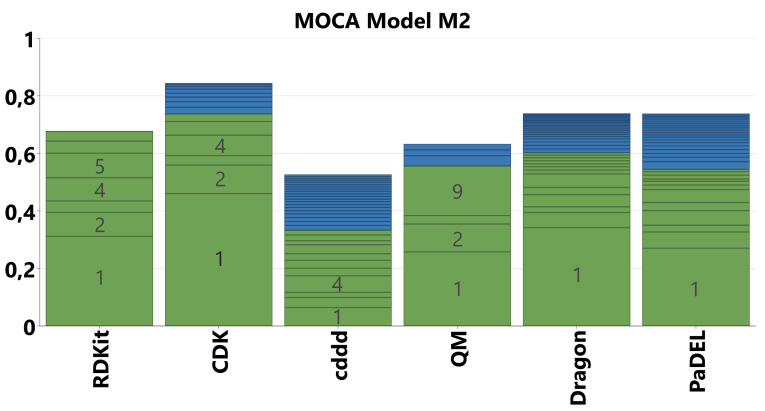
Overview of explained variance (R2) for the MOCA model M2 on the ChemGPS data set with 6 descriptor blocks. Segmentation reflects explained variance per joint (green) and unique (blue) component. Numbers of the major components are displayed in grey.

For the conventional descriptors we fitted a new MOCA model on the blocks RDKit, CDK, QM, PaDEL, Dragon, and including cddd as a new type of descriptor with similar properties like fingerprints in terms of number of components and explained variance. In total, this model (M2) was constructed on 6181 variables, separated into 6 blocks. The overview of explained variance (R2) is presented in Figure 4.

The general trends of model M1 were preserved: The first component is globally joint and explains a major part (up to 46 % for CDK) of the variance in each block. It is related to molecular size, since many descriptors in the conventional packages are size‐dependent, e. g. topological descriptors counting atoms or bonds. The second component is related to aromaticity and flexibility, e. g. the number of aromatic rings or the fraction of sp^3^‐hybridized carbon atoms. Qualitatively, the first components resemble the ones of the original ChemGPS PCA model,^[24]^ although there is some entanglement, as can be shown with bivariate correlation analysis (see Supporting Information). For example, the scores of the first joint component of M2 and the first principal component of the original ChemGPS PCA model have a correlation coefficient exceeding 0.95.

Interestingly, Winter et al.^[4]^ observed similar trends in the PCA space of the cddd descriptors on their original data set. The first and second principal components from the cddd space are highly correlated to molecular weight and logP, respectively. These cddd descriptors are completely data‐driven based on SMILES input strings and not biased by the selection of descriptors or descriptor packages.

**Figure 5:**
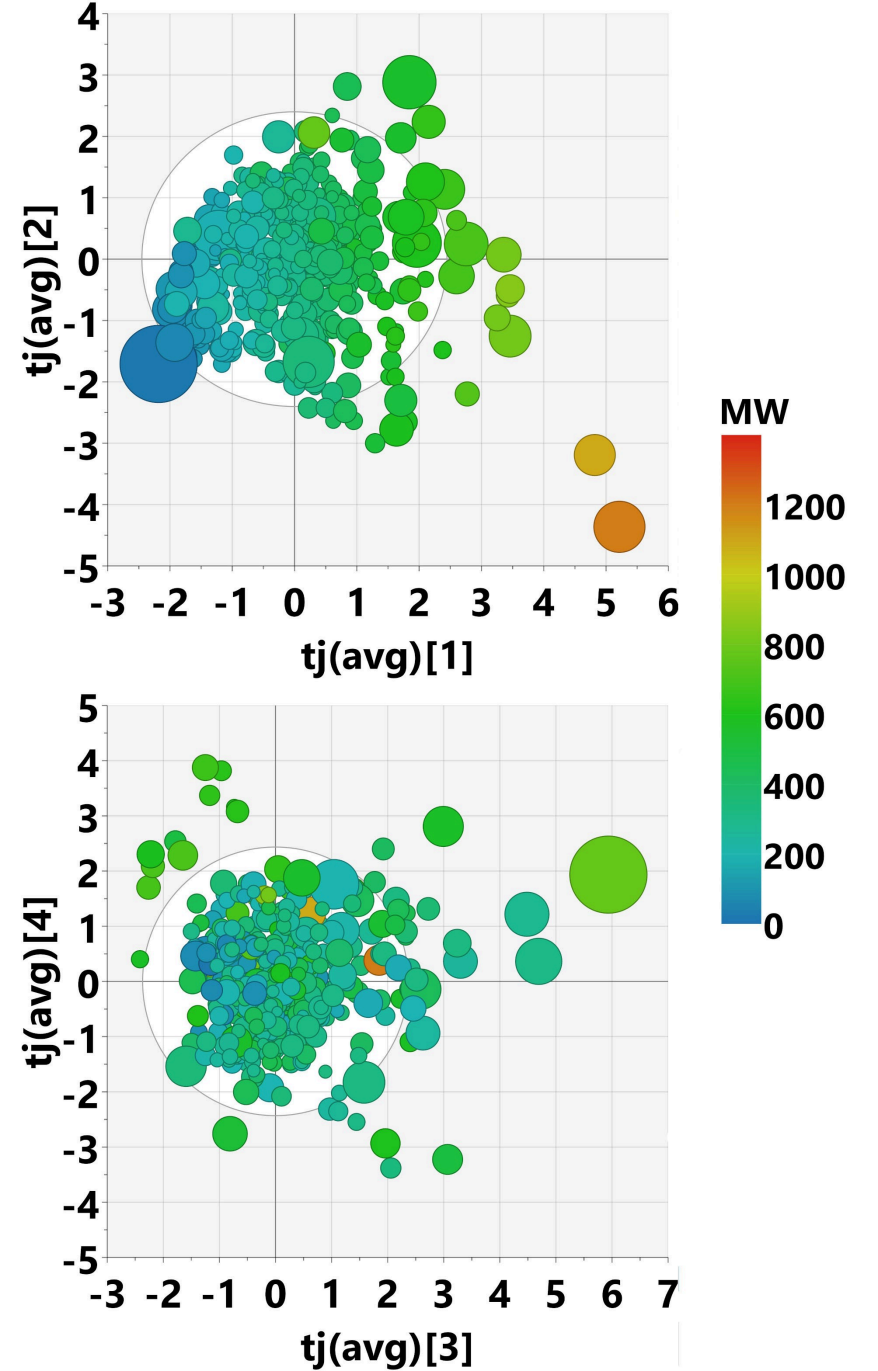
Scores plot for the first 4 components of MOCA model M2, colored by molecular weight. The size of the circle represents concordance of the individual descriptor blocks. A large circle means high deviations between blocks.

The scores plots for the first 4 components of model M2 are shown in Figure 5. The size of the circles indicates how much the scores differ between the single blocks. This plot can be used to drill down into deeper analysis for compounds of interest. For example, we identified a polyacetylene with large differences in scores between the blocks. Its large conjugated π‐system causes special electronic properties, which are reflected in higher levels of theory, but overlooked by many topological descriptor types. The two extreme molecules on the bottom right quadrant of the plot represent two oligopeptides. However, despite their high numerical score values in the first two components, they do not distort the model.

**Figure 6:**
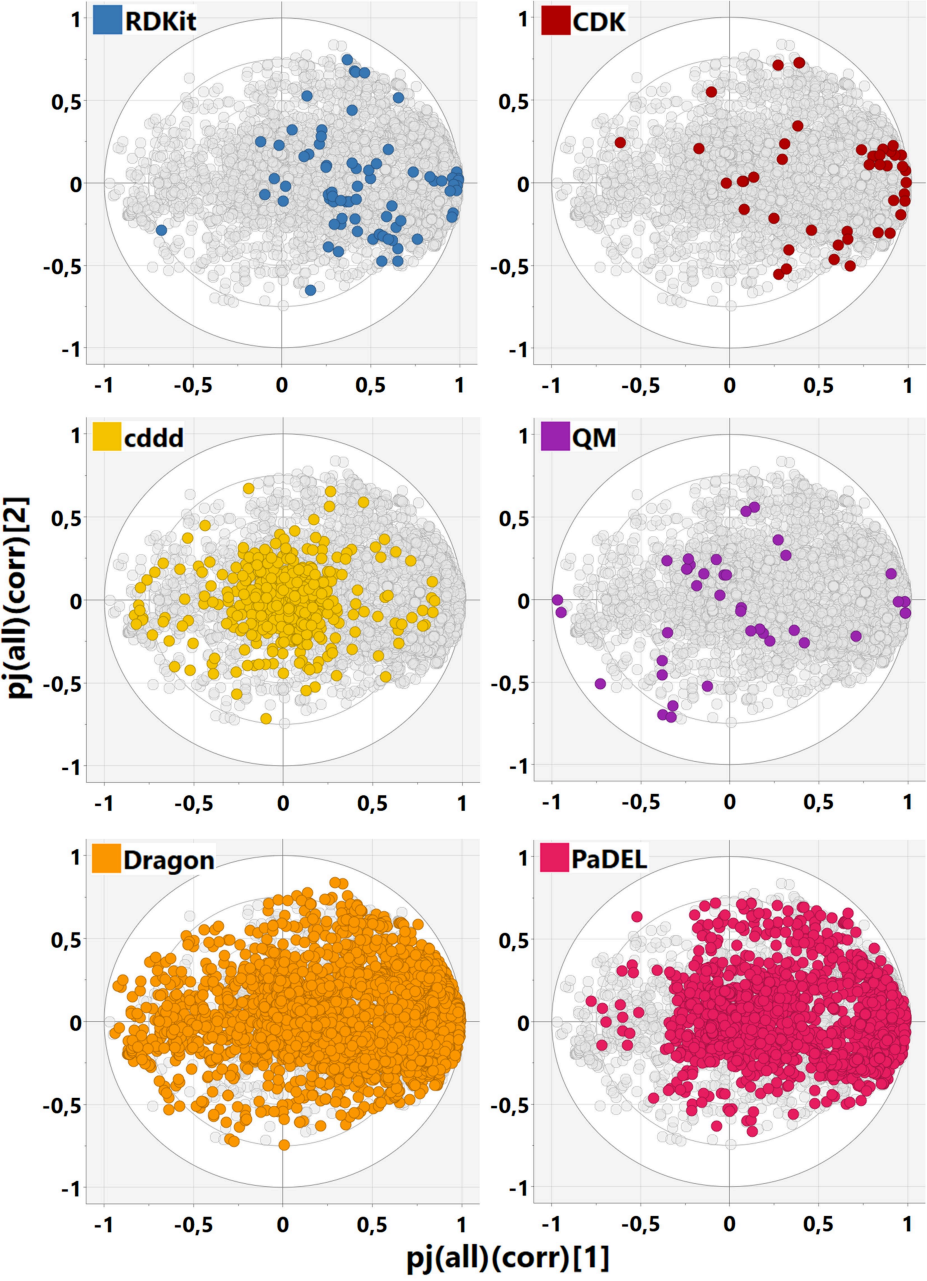
Loadings plot for MOCA model M2, highlighting the blocks separately.

The loading plots for the first two components are presented in Figure 6. Many of the CDK descriptors are strongly and mostly related to molecular size (joint component 1), e. g. the heavy atoms count and the bonds count. In contrast, the cddd descriptor loadings are spread relatively evenly, describing combinations of components 1 and 2 as well. Many are clustered at the origin but contribute to higher components instead. This is due to the design of the embedded cddd space, which is constructed based on a dimensionality reduction technique and therefore does not over‐represent particular molecular features like the molecular size. Interestingly, the QM descriptors include some loading values, which are unlike the bulk of the other descriptors, e. g. hardness (HOMO/LUMO energy difference), H_ring (COSMO‐RS energy correction due to ring atoms), or H_vdW (COSMO‐RS van‐der‐Waals energy term). These descriptors could potentially add valuable information for predictive or descriptive tasks, that cannot be replaced with the other descriptor types.

One of the main goals of our analysis is the detection of redundancy in the descriptor data, such that descriptors could be removed from the data table prior to predictive modelling tasks, to reduce the risk of spurious chance correlations. From the above analysis it becomes clear that a large fraction of descriptors is related to molecular size and can be removed. Alternatively, the degrees of freedom can be reduced by dimensionality reduction techniques like PCA or hierarchical modelling. However, hierarchical approaches miss out on the opportunity to align multi‐block components to identify more subtle commonalities.

Additionally, we wanted to test whether whole packages could be removed to reduce the computational and manual effort for descriptor calculation. The obvious way of block‐wise elimination is time consuming and can be misleading – e. g. removal of the QM block does not alter the R2 values of the remaining blocks but removes a significant amount of unique information (see Supporting Information Section 3). We therefore propose the fraction of explained variance by joint components (R2Xj) in the full model as a first step selection criterion: the information from blocks with high R2Xj (large green bars in the R2 overview plot) is redundant and can be approximated by the other packages. The explained variance by unique components indicate structure in the data of a single block, which is unique for this block. The remaining fraction of the unexplained variance might contain unique information as well. We compiled a summary of this information for model M2 in Table 3. In addition to the pure R2Xj values, we propose a metric that takes the maximum correlation to the joint component of any other block into account as a weighting factor. With this redundancy metric R (equation (1)) we estimate how well information from the *target* block is covered by the joint component of all the other blocks *A*.

(1) $$R_{T}\;=\sum_{j}{R2X}_{j,T}{}^{*}max\left(\left|correl\left(t_{j,A}\;,\;t_{j,T}\right)\right|\right)$$

**Table 3:** Comparison of descriptor packages and their explained variance by joint components in model M2.

	RDKit	CDK	cddd	QM	Dragon	PaDEL
number of descriptors^a^	74	45	512	40	3868	1642
explained variance by joint components, R2X	0.68	0.74	0.33	0.56	0.60	0.54
explained variance by unique components, R2Xu	0.00	0.11	0.19	0.08	0.14	0.19
redundancy R_T_	0.65	0.73	0.32	0.52	0.59	0.54
costs (licenses, labour time)^b^	5	5	5	1	3	4
explainability (interpretation, reversibility)^b^	3	3	3	4	2	2

^a^ After removal of constant values. ^b^ Personal choice of the authors. Criteria and scores depend on the use case and reflect personal preferences. Other criteria might be added; scores range from 0 (worst) to 5 (best).

The highest R2Xj and redundancy values were found for the RDKit and CDK blocks. One of these packages can be removed without losing much information. Additionally, other factors like license costs, computation time, or explainability of the descriptors can be taken into consideration. Of course, these “soft” criteria depend on the use case and the availability of software tools and should be re‐assessed for each project individually. Nevertheless, we provided suggestions based on personal preferences in Table 3.

Another important criterion is the total number of descriptors in one package. Exclusion of larger packages reduces the final number of descriptors most efficiently. On the other hand, larger packages tend to contribute more unique variance at the same time. For a fairer comparison, larger packages could be split into sub‐blocks of comparable size based on their underlying theoretical concept or molecular properties. However, this is beyond the scope of this article and currently limited by computational costs, which scale more than linearly with the number of blocks. For our example project below, i. e. analysis of the Pesticides data set, we decided to leave out the CDK block due to its high redundancy value.

Another question we pursued, was whether the computationally expensive and labor‐intensive QM descriptors add value? MOCA model (M2) constructed 3 unique components for this block. Prominent descriptors with high loadings into these components are for example hydrogen bond donor capability, polarizability, dipole moment, and the sigma profiles of the COSMO‐RS solvation model. These variables describe the intermolecular interactions of the molecules with their environment and apparently contain unique information, which is not covered by the other descriptor packages. If the modelled properties are expected to depend on these features, it might be worth the effort to include the QM descriptors.

### 3.3 Analysis of Descriptor Blocks for an Example Project Including Biological Data (Pesticides Data Set)

**Figure 7:**
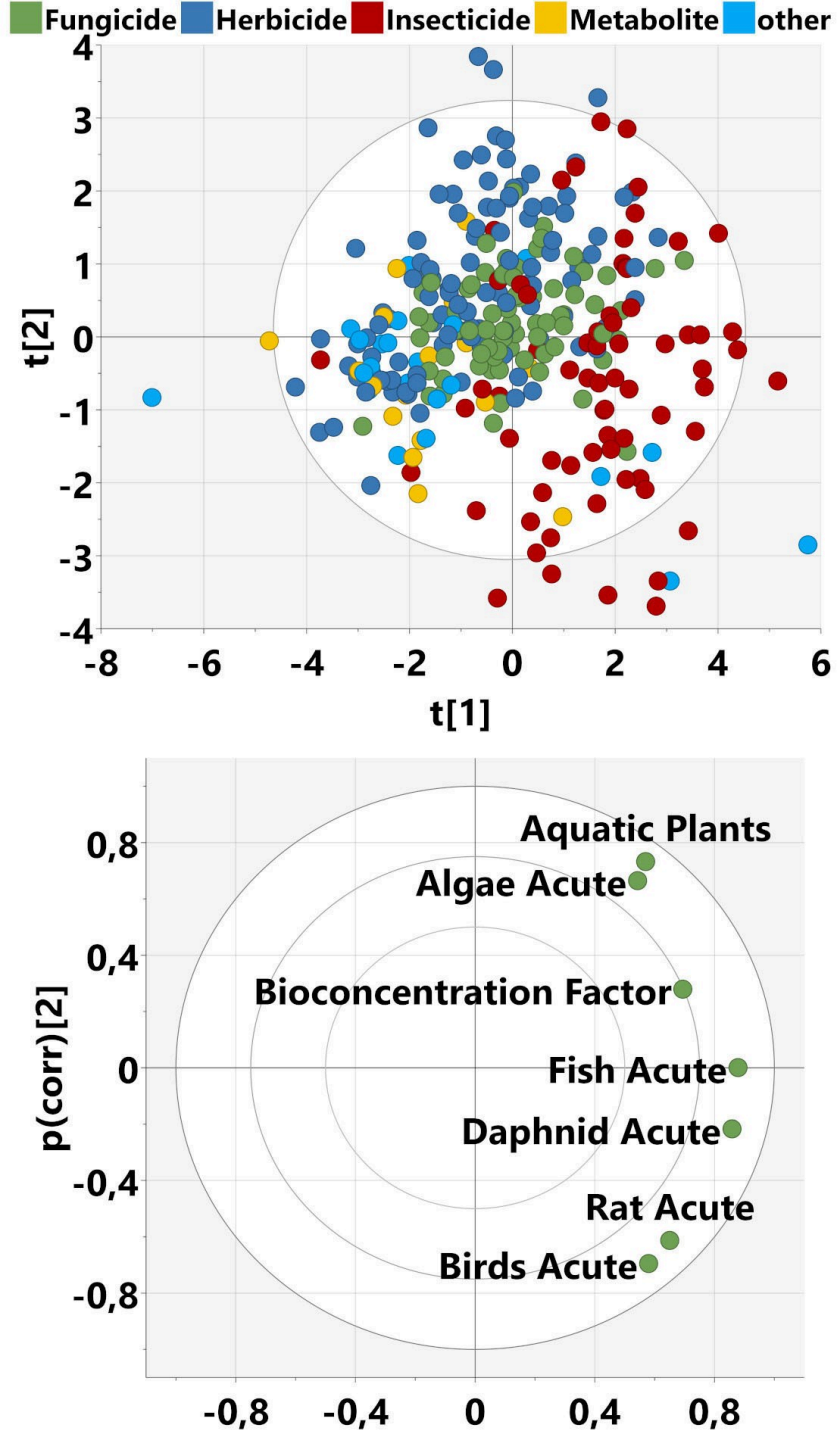
Principal Component Analysis (PCA) on the bio block of the Pesticides data set. Top: scores plot. Bottom: loadings plot. PC1 and PC2 represent 51 % and 21 % explained variance, respectively.

We applied our learnings from the ChemGPS data set to the Pesticides data set as an example project, which is relevant for crop protection chemistry and environmental safety. In addition to the calculated molecular descriptors, this set includes biological data in the form of 7 ecotoxicological study endpoints (see Table 1). Figure 7 shows the scores and loadings of a principal component analysis of these endpoints. Overall, the explained variance (R2X) of the PCA model with two components is 0.7, which means there are trends among the experimental variables. This can be expected due to known inter‐species correlations reported before (see references[^47^, ^48^, ^49^, ^50^]). The highest correlation coefficient we found in our data set is between fish and daphnid with 0.8. The first component indicates higher toxicity towards all species and a higher tendency for bioaccumulation. The second component differentiates between algae and aquatic plants on the one hand (higher values) versus rats and birds on the other hand (lower values). It can also be seen from the scores plot (Figure 7, top) that insecticides have a higher tendency to be toxic for the investigated species than other indications (insecticides are in the right half of the scores plot). Herbicides are often more toxic to aquatic plants and algae as compared to rats and birds (top quadrants). Fungicides do not show a clear prevalence towards any species (center of the scores plot).

Thus, the PCA confirms the common assumption that the different property profiles of insecticides on one side and herbicides and fungicides on the other side reflect their different toxicities. Ideally, herbicides are systemic, penetrating into plants and being transported to their target proteins. This requires them to be soluble in water. On the other hand, insecticides need to be protected from the efficient metabolic capacity of insects, leading to a reduced number of aromatics, an increased number of halogens and consequently a loss of systemicity. Hence, on average insecticides have a higher lipophilicity and a lower water solubility than herbicides and fungicides.^[51]^ The tendency of herbicides to be more toxic to aquatic plants and algae than to rats and birds can be explained by the fact that important herbicidal modes of action like the photosynthesis system are not present in rats or birds.

**Figure 8:**
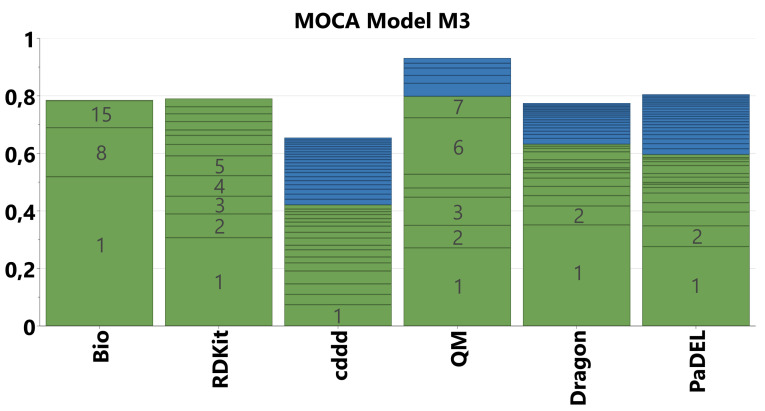
Overview of explained variance (R2) by blocks for MOCA model M3. Segmentation reflects explained variance per joint (green) and unique (blue) component. Numbers of the major components are displayed in grey.

Moving forward to the MOCA analysis of the Pesticides set, we obtained a very similar model compared to model M1 using all descriptors (not shown), indicating transferability of the results. This is mainly because both sets cover a relatively broad and similar molecular property space. We thus reduced the model to 5840 descriptors from the 5 software packages we identified as most relevant for the ChemGPS data set. The structure of the model is qualitatively similar to the previous model M2. However, using default settings, the model did not include any components from the biological block. This is due to the very small size and the noisiness of the bio block. Since we were particularly interested in the correlations with the bio block, we relaxed the strictness of the model (loading length limit of −0.2 instead of −0.03), such that the joint components are constructed also from less strictly correlated features and include smaller blocks as well. We thus obtained model M3, which contains 3 joint components with contributions from the bio set (Figure 8). The intra‐component correlation between the bio block and the other blocks for these 3 components is around 0.6 to 0.7 (Pearson's correlation coefficient r^2^), indicating that the bio block is indeed related to the other blocks (see Table S2 and score correlation matrix in the Supporting Information).

In total, the joint components cover an explained variance of nearly 80 % of the bio block, without any unique bio components (Figure 8), which demonstrates that linear QSAR models should be able to capture a large fraction of the variance in the experimental data. For the descriptor blocks the coverage of explained variance is similar to models M1 and M2 on the ChemGPS data set.

The first model component is globally joint and strongly related to molecular size and overall biological activity, in line with the ChemGPS MOCA models M1/M2 and the PCA on the biological variables. Descriptors with high loadings are for example molecular volume, surface area or number of (heavy) atoms. All biological variables have positive loadings (0.5 to 0.9). This component has by far the highest coverage of explained variance across all blocks, with 0.3 for the conventional and QM blocks and 0.5 for the bio block. Typically, these size descriptors appear to be important features also for many other QSAR models for biological activities (author‘s experience; see also^[52]^). One mechanistic explanation is their relation to thermodynamic or physico‐chemical properties like solubility (bigger molecules must displace more solvent molecules and are therefore less soluble), higher lipophilicity, or larger contact surfaces for molecular interactions. From the bio block, the loadings of the aquatic organisms are higher than for birds and rats. Thus, the component confirms that molecular size is especially related to higher biological activity in aquatic organisms.

**Figure 9:**
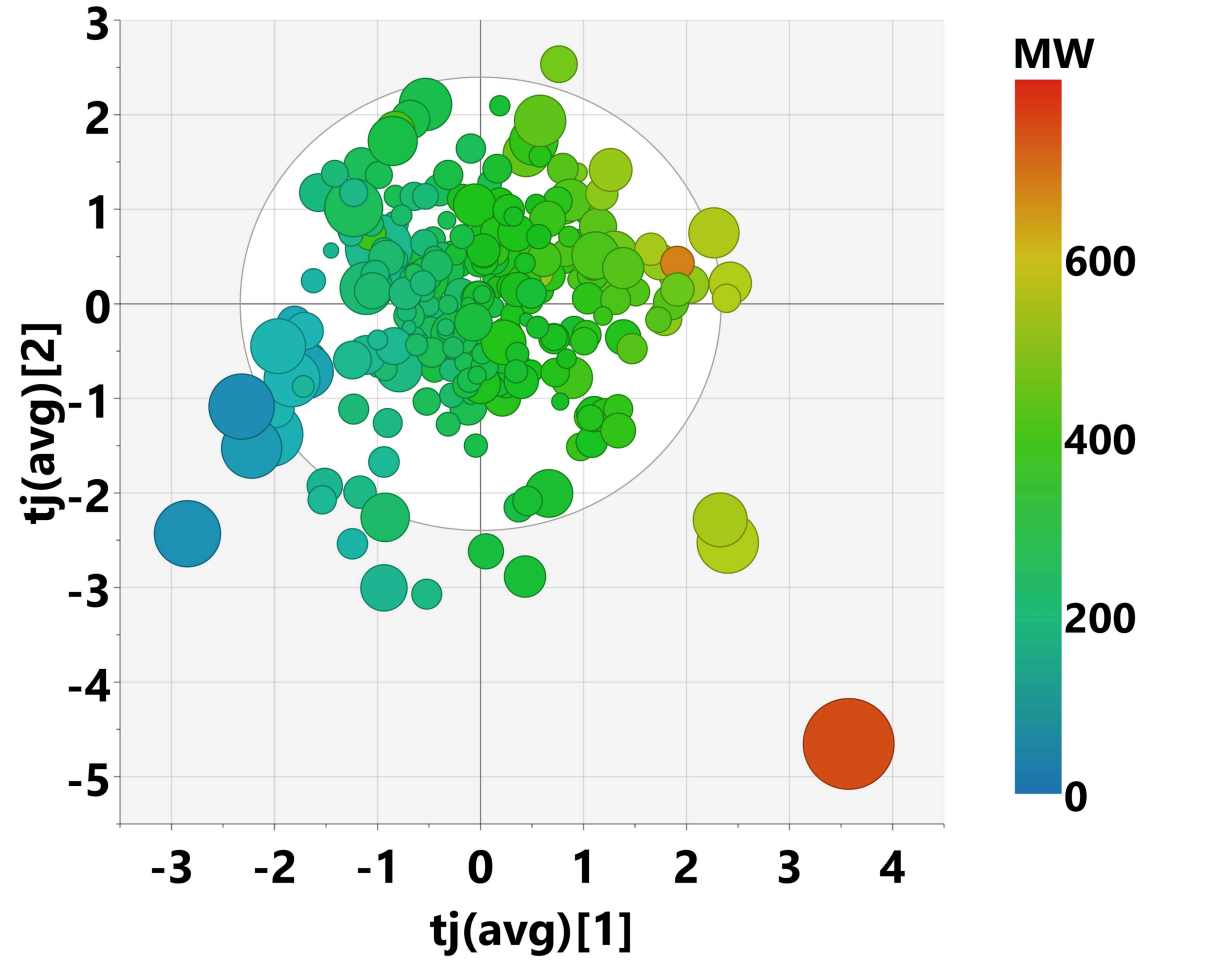
Scores plot of joint components 1 and 2 of the Pesticides MOCA model M3, colored by molecular weight. The size of the markers indicates differences between individual blocks.

The second joint component is related to bond saturation: compounds with only single bonds vs. compounds with many aromatic rings or double bonds. It is not joined with the bio block. The scores plot of the two first joint components is shown in Figure 9.

**Figure 10:**
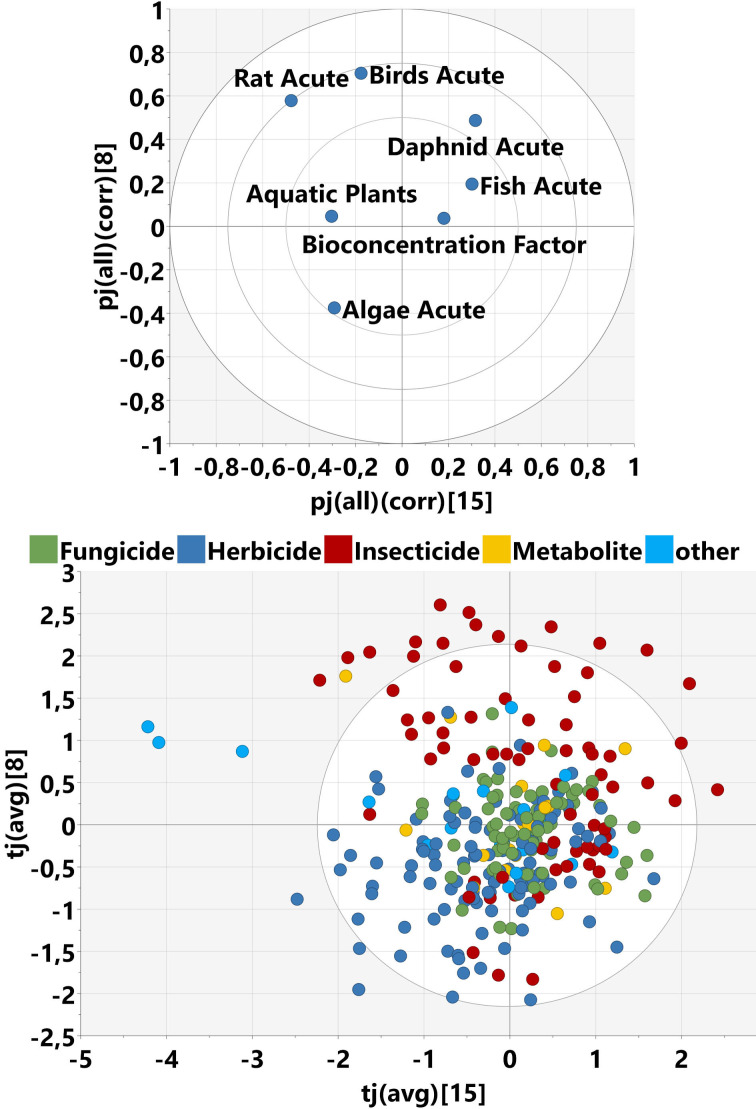
Top: Loadings plot of joint components 8 and 15 of the Pesticides MOCA model M3, showing only the bio variables. Bottom: Scores plot for this model, colored by indication.

The other two components joint with the bio block (number 8 and 15) cover only a small fraction of explained variance in the bio block (0.17 and 0.09, respectively) and even less for the other blocks (from 0 to 0.02). We plotted the loadings from the bio variables and the scores side by side (Figure 10). This allows easy comparison because molecules on one end of the scores plot have high loadings from variables on the same end of the loadings plot. The loadings from the organisms are clustered in a similar way as in the PCA model above (birds/rats vs fish/daphnid vs aquatic plants/algae) indicating a differentiation between the organisms (Figure 10, top). The scores plot (Figure 10, bottom) shows that component 8 separates insecticides (top quadrants) from herbicides (bottom quadrants), whereas for component 15 there are 3 outlier molecules (left) sticking out from the rest: difethialone, flocoumafen, and difenacoum. These are active ingredients of rodenticides and thus show high toxicity towards rats. The loadings of the descriptors are generally low for both components, with a maximum of 0.43 (P‐117_Dragon) and 0.34 (cddd_248) for components 8 and 15, respectively. We did not identify any outstanding molecular features, which could provide a clear explanation for the different biological activities from this analysis.

### 3.4 Towards Predictive Models

In preparation of developing predictive QSAR models, we were also interested in a systematic approach to check for correlations with the experimental bio block. A simple correlation analysis between single bio variables and the most correlated descriptors has two major disadvantages: a) high levels of noise, i. e. we would find many descriptors which are only correlated by chance due to the sheer number, and b) correlation to *differences* between species would be overlooked. Instead, we analyzed the joint components of the MOCA model M3, which are of much lower dimensionality (lower risk of spurious chance correlation), provide a rough mechanistic interpretation, and can represent differences between species like components 8 and 15.

We calculated predictivity scores for each joint component as the product of the explained variance in the bio block (R2X_j,T_) and the correlation coefficient between score vectors from the bio block (t_j,T_) and the descriptor block (t_j,A_), as obtained from the score correlation matrix. We propose to use the sum over all components as overall predictivity score P_A_ for a particular block A (equation 2.

(2) $$P_{A}\;=\sum_{j}{R2X}_{j,T}{}^{*}\left|correl\left(t_{j,A}\;,\;t_{j,T}\right)\right|$$

For the bio block the correlation is 1 and the score value thus corresponds to the explained variance. We summarized our analysis in Table 4. The predictivity score is an indicator for the fraction of explained variance in the whole target block, without having to build a separate model for each block. We confirmed this approach by building OPLS regression models for each descriptor block (last row in Table 4). The predictivity scores agree very well with the explained variance R2Y(bio) of the OPLS models.

**Table 4:** Correlation metrics for individual blocks and components with respect to the target bio block in model M3.

component	bio	RDKit	cddd	QM	Dragon	PaDEL
tj[1]	0.52	0.32	0.35	0.32	0.37	0.35
tj[8]	0.17	0.07	0.13	0	0.13	0.12
tj[15]	0.09	0	0.07	0	0	0.06
pred. score P_A_	0.78	0.40	0.54	0.32	0.50	0.54
R2Y(bio) from OPLS 2‐block, 3 pred. LV	–	0.38	0.54	0.27	0.46	0.46

Component 1 (“overall ecotoxicity”) is joint over all blocks. Only two blocks contribute to all 3 joint components: cddd and PaDEL, with cddd being the block with a smaller number of variables and higher R2Y in the OPLS model. Thus, we conclude that the cddd descriptors are the most versatile individual block for modeling this ecotoxicological data set. This conclusion is corroborated by the results of a detailed investigation of PLS models of both the full target bio block and each target with each descriptor block (Supporting Information, Table S3).

Heading towards predictive models, the attempt to cover all 7 ecotoxicological endpoints at once is probably too difficult a task. However, it can be beneficial to address related endpoints in a multi‐task approach, which can improve predictivity and robustness of the models.^[53]^ Based on Figure 7, suitable pairs are fish and daphnid, algae and aquatic plants, or birds and rats. Indeed, models based on fish and daphnid toxicity have already been reported.[^54^, ^55^] Exemplarily, we built a PLS model for the fish and daphnid endpoints. Without any further optimization the explained variance R2Y of 0.782 (cross‐validated 0.523) indicates a good starting point for model development. Reduction of the descriptors to the cddd block leads to an insignificant drop in R2Y to 0.758 (cross‐validated 0.548). For the QM block this drop was more severe, to 0.506 (cross‐validated 0.383). As might be expected from the first component of the MOCA model being related to the molecular size, using only the molecular weight as single X variable can already explain 0.271 of the variance (cross‐validated 0.251). Overall, these findings represent a promising starting point for the development of predictive QSAR models. However, a detailed description of model development is out of scope for this article and subject to future work.

## 4 Conclusions

Data tables for multivariate analysis or machine learning projects are often naturally organized in blocks of data, although this structure frequently is not exploited. We used Multiblock Orthogonal Component Analysis as a navigation tool to explore such a data table for QSAR modelling tasks, where each block contains chemical representations in the form of molecular descriptors calculated with frequently used software packages.

Applied to the set of ChemGPS reference molecules, which were selected to represent chemical space in a broad and robust way for principal component models, we were able understand more about the nature of the descriptor packages and (re‐)discovered trends in the underlying data: A large fraction of the conventional molecular descriptor types are correlated with molecular size, lipophilicity, aromaticity, and flexibility, which have consistently been identified as important discriminators between molecules and trends in the data, see e. g. the original ChemGPS publication,^[23]^ deep learning projects,^[4]^ and many QSAR models.^[52]^ Care must be taken during QSAR model development to not let molecular size, which is overly represented in descriptor space, take away attention from other important properties, especially when using automated approaches. This can be achieved by removing correlated descriptors from the data table or with hierarchical modelling methods as dimensionality reduction techniques.

Additionally, MOCA was helpful for deciding, which software packages could potentially be removed from the project on a general level, i. e. without peeking at predictive performance for a specific task. We proposed a redundancy score and identified the CDK and RDKit KNIME nodes as candidates for removal without losing much information. Also, the additional descriptors in a new release of the alvaDesc software and an internal set of descriptors turned out to not add significant amounts of new information. On the other hand, the recently developed cddd descriptors, which were derived from a deep learning translation task, seem to have promising potential, which can be seen at one glance in the R2 plot: high orthogonality (low R2X values per component), covering most joint components (and thus other descriptor packages), adding unique information, and relatively low dimensionality.

We compiled a second data set consisting of crop protection chemicals with a relatively dense availability of 7 ecotoxicological endpoints. Some trends exist within this data set, e. g. a high correlation between toxicity towards fish and daphnids. We used MOCA to analyse correlations between these biological activities and the molecular descriptor blocks. Almost 80 % of the variance in the bio block was attributed to joint components with the descriptor blocks, which indicates that (linear) QSAR models should be feasible. The model structure is comparable to the model on the ChemGPS set. The first joint component has again very high fractions of explained variance across all blocks and is related to molecular size and the overall ecotoxicity, especially towards aquatic organisms. Two other joint components of the bio block describe more specific properties of the data set like a differentiation between species or influence of rodenticides. These trends can then be linked to molecular descriptors in a more detailed analysis leading to mechanistic interpretations where feasible.

In preparation of predictive modelling, we propose a predictivity score based on the joint components of the MOCA model, to indicate the most predictive descriptor blocks for the biological properties. The cddd descriptors appeared to be most suitable, considering the correlation and coverage of joint components as well as the number of descriptor variables.

Overall, we consider MOCA a useful tool to explore data sets for QSAR modelling block‐wise by groups of descriptors. One single MOCA model can already indicate multiple important trends regarding similarity of descriptor packages, molecular properties, clustering or outlier molecules, and predictivity of descriptor sets.

## Disclosure Statement

SS and MS are employees of Bayer AG, a manufacturer of pharmaceuticals, agricultural, and consumer health chemicals. LE is employee of Sartorius Stedim Data Analytics AB, a distributor of data analytics software.

## Conflict of interest

SS and MS are employees of Bayer AG, a manufacturer of pharmaceuticals, agricultural, and consumer health chemicals. LE is employee of Sartorius Stedim Data Analytics AB, a distributor of data analytics software.

5

## Supporting information

As a service to our authors and readers, this journal provides supporting information supplied by the authors. Such materials are peer reviewed and may be re‐organized for online delivery, but are not copy‐edited or typeset. Technical support issues arising from supporting information (other than missing files) should be addressed to the authors.

Supporting Information

## Data Availability

The data tables containing the calculated descriptors as well as 3D molecular structures are openly available via figshare: https://doi.org/10.6084/m9.figshare.c.5446677. SIMCA® is available in a demo‐version, so the interested reader can reproduce our results.

## References

[1] R. Todeschini , V. Consonni , Molecular Descriptors for Chemoinformatics, Wiley-VCH, Weinheim, 2009.

[2] A. Mauri , V. Consonni , R. Todeschini in Handbook of Computational Chemistry, Springer International Publishing, Cham, 2017.

[3] K. V. Chuang , L. M. Gunsalus , M. J. Keiser , J. Med. Chem. 2020, 63, 8705. 32366098 10.1021/acs.jmedchem.0c00385

[4] R. Winter , F. Montanari , F. Noé , D.-A. Clevert , Chem. Sci. 2019, 10, 1692. 30842833 10.1039/c8sc04175j PMC6368215

[5] S. Riniker , G. A. Landrum , J. Cheminf. 2013, 5, 26. 10.1186/1758-2946-5-26 PMC3686626 23721588

[6] F. Grisoni , V. Consonni , R. Todeschini in Computational Chemogenomics (Ed.: J. B. Brown ), Humana Press, New York, 2018, pp. 171–209.

[7] T. Stepišnik , B. Škrlj , J. Wicker , D. Kocev , Comput. Biol. Med. 2021, 130, 104197. 33429140 10.1016/j.compbiomed.2020.104197

[8] S. Wold , N. Kettaneh , K. Tjessem , J. Chemom. 1996, 10, 463.

[9] A. Berglund , M. C. de Rosa , S. Wold , J. Comput.-Aided Mol. Des. 1997, 11, 601. 9491352 10.1023/a:1007983320854

[10] J. A. Westerhuis , T. Kourti , J. F. MacGregor , J. Chemom. 1998, 12, 301.

[11] K. Tøndel , J. O. Vik , H. Martens , U. G. Indahl , N. Smith , S. W. Omholt , Chemom. Intell. Lab. Syst. 2013, 120, 25.

[12] J. Gabrielsson , H. Jonsson , C. Airiau , B. Schmidt , R. Escott , J. Trygg , J. Chemom. 2006, 20, 362.

[13] A. Höskuldsson , K. Svinning , J. Chemom. 2006, 20, 376.

[14] S. Hassani , H. Martens , E. M. Qannari , M. Hanafi , A. Kohler , Chemom. Intell. Lab. Syst. 2012, 117, 42.

[15] M. Tenenhaus , V. E. Vinzi , Y.-M. Chatelin , C. Lauro , Comput. Stat. Data Anal. 2005, 48, 159.

[16] B. Liquet , P. Lafaye de Micheaux , B. P. Hejblum , R. Thiébaut , Bioinformatics 2016, 32, 35. 26358727 10.1093/bioinformatics/btv535

[17] M. Bylesjö , D. Eriksson , M. Kusano , T. Moritz , J. Trygg , The Plant Journal 2007, 52, 1181. 17931352 10.1111/j.1365-313X.2007.03293.x

[18] E. F. Lock , K. A. Hoadley , J. S. Marron , A. B. Nobel , Annals Appl. Stat. 2013, 7, 523. 10.1214/12-AOAS597 PMC3671601 23745156

[19] M. Schouteden , K. van Deun , S. Pattyn , I. van Mechelen , Behavior Research Methods 2013, 45, 822. 23361416 10.3758/s13428-012-0295-9

[20] T. Löfstedt , J. Trygg , J. Chemom. 2011, 25, 441.

[21] T. Löfstedt , D. Hoffman , J. Trygg , Anal. Chim. Acta 2013, 791, 13. 23890602 10.1016/j.aca.2013.06.026

[22] T. Skotare , R. Sjögren , I. Surowiec , D. Nilsson , J. Trygg , J. Chemom. 2020, 34, e3071.

[23] L. Eriksson , E. Johansson , N. Kettaneh-Wold , J. Trygg , C. Wikstrom , S. Wold , Multi- and Megavariate Data Analysis, 2nd Edition, Umetrics AB, Umea, Sweden, 2006.

[24] T. I. Oprea , J. Gottfries , J. Comb. Chem. 2001, 3, 157. 11300855 10.1021/cc0000388

[25] K. A. Lewis , J. Tzilivakis , D. J. Warner , A. Green , Human and Ecological Risk Assessment: An International Journal 2016, 22, 1050.

[26] RDKit: Open-source cheminformatics, *available from http://www.rdkit.org*, **2020**.

[27] C. Steinbeck , Y. Han , S. Kuhn , O. Horlacher , E. Luttmann , E. Willighagen , J. Chem. Inform. Comput. Sci. 2003, 43, 493. 10.1021/ci025584y PMC4901983 12653513

[28] M. R. Berthold , N. Cebron , F. Dill , T. R. Gabriel , T. Kötter , T. Meinl , P. Ohl , C. Sieb , K. Thiel , B. Wiswedel , in Studies in Classification, Data Analysis, and Knowledge Organization (GfKL), Springer, 2007.

[29] Sybyl Version X 2.1 – *Discovery Software for Computational Chemistry and Molecular Modelling, including UNITY fingerprint tools* **2004**.

[30] TURBOMOLE V7.0.1, a *development of University of Karlsruhe and Forschungszentrum Karlsruhe GmbH, 1989-2007, TURBOMOLE GmbH, since 2007; available from http://www.turbomole.com* **2016**.

[31] F. Eckert , A. Klamt , AIChE J. 2002, 48, 369.

[32] F. Eckert , A. Klamt , COSMOtherm, Version C3.0, Release 16.01 2015.

[33] Dragon v7.0: *Software for Molecular Descriptor Calculation* **2017**.

[34] C. W. Yap , J. Comput. Chem. 2011, 32, 1466. 21425294 10.1002/jcc.21707

[35] A. Mauri, in *Ecotoxicological QSARs. Methods in Pharmacology and Toxicology (Ed.: K. Roy) Humana, New York, NY*, **2020**, pp. 801–820.

[36] SIMCA 16, *available from www.sartorius.com/umetrics* **2020**.

[37] J. E. Jackson , A User's Guide to Principal Components, John Wiley, New York, 1991.

[38] S. Wold , K. Esbensen , P. Geladi , Chemom. Intell. Lab. Syst. 1987, 2, 37.

[39] L. Eriksson , T. Byrne , E. Johansson , J. Trygg and C. Wikström , Multi- and Megavariate Data Analysis – Basic Principles and Applications, 3rd Ed. , Umetrics , Malmö, 2013.

[40] S. Wold , M. Sjöström , L. Eriksson , Chemom. Intell. Lab. Syst. 2001, 58, 109.

[41] L. Eriksson , J. Trygg , S. Wold , J. Chem. 2014, 28, 332.

[42] J. Trygg , S. Wold , J. Chemom. 2002, 16, 119.

[43] S. Wiklund , E. Johansson , L. Sjöström , E. J. Mellerowicz , U. Edlund , J. P. Shockcor , J. Gottfries , T. Moritz , J. Trygg , Analyt. Chem. 2008, 80, 115. 18027910 10.1021/ac0713510

[44] L. Eriksson , J. Rosén , E. Johansson , J. Trygg , Mol. Inf. 2012, 31, 414. 10.1002/minf.201200158 27477460

[45] H. L. Morgan , J. Chem. Doc. 1965, 5, 107.

[46] D. Rogers , M. Hahn , J. Chem. Inf. Mod. 2010, 50, 742. 10.1021/ci100050t 20426451

[47] X. J. Zhang , H. W. Qin , L. M. Su , W. C. Qin , M. Y. Zou , L. X. Sheng , Y. H. Zhao , M. H. Abraham , Sci. Total Environ. 2010, 408, 4549. 20673582 10.1016/j.scitotenv.2010.07.022

[48] S. Raimondo, D. N. Vivian, M. G. Barron, *Web-based interspecies correlation estimation (Web-ICE) for acute toxicity: user manual; Office of Research and Development. US Environmental Protection Agency, Gulf Breeze, FL, USA* **2010**.

[49] L. Y. Fan , D. Zhu , Y. Yang , Y. Huang , S. N. Zhang , L. C. Yan , S. Wang , Y. H. Zhao , Ecotoxicol. Environ. Saf. 2019, 177, 25. 30954009 10.1016/j.ecoenv.2019.03.111

[50] K. Bouhedjar , E. Benfenati , A. K. Nacereddine , SAR QSAR Environ. Res. 2020, 31, 785. 32878491 10.1080/1062936X.2020.1810770

[51] E. D. Clarke , J. S. Delaney , CHIMIA Internat. J. Chem. 2003, 57, 731.

[52] L. Mamy , D. Patureau , E. Barriuso , C. Bedos , F. Bessac , X. Louchart , F. Martin-laurent , C. Miege , P. Benoit , Crit. Rev. Environ. Sci. Technol. 2015, 45, 1277. 25866458 10.1080/10643389.2014.955627 PMC4376206

[53] F. Montanari , L. Kuhnke , A. ter Laak , D.-A. Clevert , Molecules 2019, 25, 44. 10.3390/molecules25010044 PMC6982787 31877719

[54] A. Kienzler , M. Halder , A. Worth , Toxicol. Environ. Chem. 2017, 99, 1129.

[55] A. Furuhama , T. I. Hayashi , H. Yamamoto , SAR QSAR Environ. Res. 2018, 29, 725. 30182748 10.1080/1062936X.2018.1513423

