# Supplementary material for: Block‐wise Exploration of Molecular Descriptors with Multi‐block Orthogonal Component Analysis (MOCA)
Source: Mol Inform. 2021 Dec 8;41(5):2100165. doi: 10.1002/minf.202100165 (PMC9285065; doi:10.1002/minf.202100165)
Supplement: Supplementary file 1 — Supporting Information [file MINF-41-0-s001.pdf]

molecular informatics

Supporting Information

Block-wise Exploration of Molecular Descriptors with Multi-block Orthogonal Component Analysis (MOCA)

Sebastian Schmidt,* Michael Schindler, and Lennart Eriksson© 2021 The Authors. Molecular Informatics published by Wiley-VCH GmbH. This is an open access article under the terms of the Creative Commons Attribution License, which permits use, distribution and reproduction in any medium, provided the original work is properly cited.

Block-wise exploration of molecular descriptors with Multi-block Orthogonal Component Analysis (MOCA)

Supporting Information

Sebastian Schmidt[1*], Michael Schindler[1], Lennart Eriksson[2*]

[1] Bayer AG, Crop Science Division, Alfred-Nobel-Str. 50, 40789 Monheim, Germany

[2] Sartorius Stedim Data Analytics AB, Östra Strandgatan 24, SE-903 33 Umeå, Sweden

Supporting Information

1. Analysis of the fingerprint blocks using the ChemGPS set 2
2. Correlation of joint components in MOCA model M2 with original ChemGPS components 3
3. Block wise elimination in MOCA model M2 on the ChemGPS data set 5
4. Correlation of joint components in model M3 on the Pesticides set 6
5. Systematic comparison of PLS models for individual blocks and ecotox endpoints 8

1. Analysis of the fingerprint blocks using the ChemGPS set

For the ChemGPS data set we analyzed the fingerprint blocks in a separate model. **Figure S1**a provides an overview of the explained variances R2 of the joint and unique components for a model built from the 4 fingerprint blocks ECFP4, unity, MACCS, and Morgan, as well as the cddd descriptor block (model M4). Most strikingly, ECFP4 and Morgan blocks don't contribute any unique components and show very similar patterns in R2. We expected this due to the very similar design of these two types of fingerprints (at their core they are both based on the same algorithm).

a)

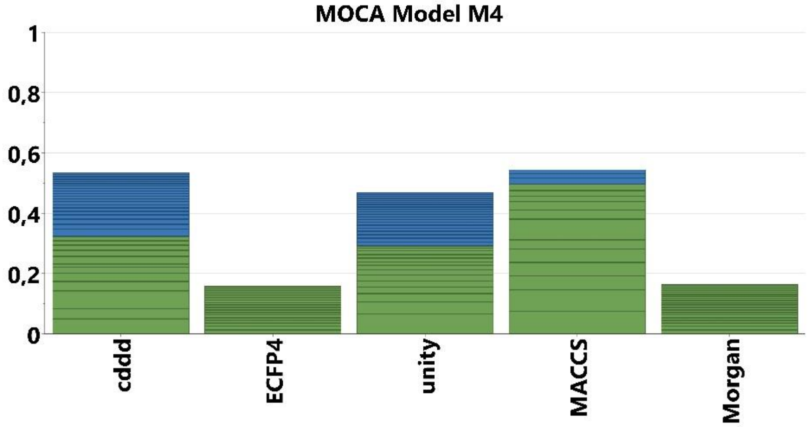

b)

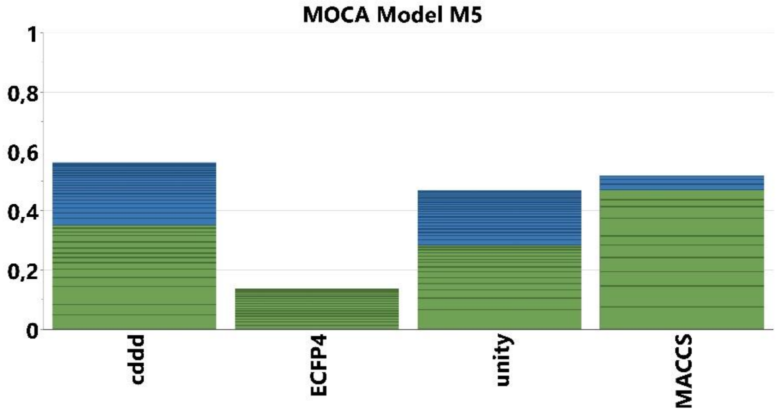

Figure S1: Overview of explained variance (R2) for the MOCA models M4 (a) and M5 (b) on the ChemGPS data set, with 5 and 4 descriptor blocks, respectively. Explained variance R2 is separated into joint components (green) and unique components (blue).

Therefore, we removed the Morgan block in the next step and built a model on the remaining 4 blocks (model M5). The total explained variance and the number of joined components did not change significantly (**Figure S1**b), neither compared to the fingerprint model with 5 blocks nor compared to the full model M1 from the main text, which demonstrates the robustness of the MOCA approach. We conclude that one of the two blocks (ECFP4/Morgan) is sufficient for modelling tasks. Moreover, all four blocks add information in a 'fine-grained' manner. The ECFP4 fingerprint is the most extreme type in this

regard, with extremely low explained variances per component, which indicates that the fingerprint bits contain a lot of orthogonal information.

2. Correlation of joint components in MOCA model M2 with original ChemGPS components

It is of interest to compare the joint scores of model M2 with the PCA model used in the original ChemGPS application. To this end, the averaged score vectors of the 13 joint components from M2 (represented by the green areas in **Figure S2**a) and the score vectors of the 4 first principal components from the ChemGPS model were combined as a new dataset. The reason for stopping at 4 principal components was there was no increase in predictive power using the fifth principal component and higher order components (**Figure S2**b).

a)

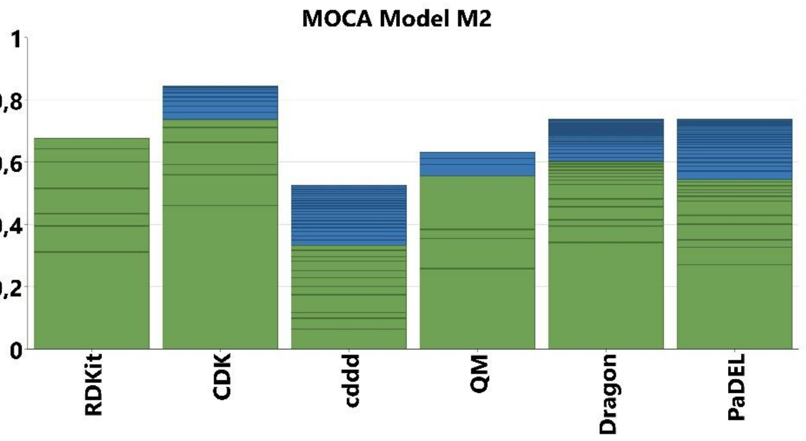

b)

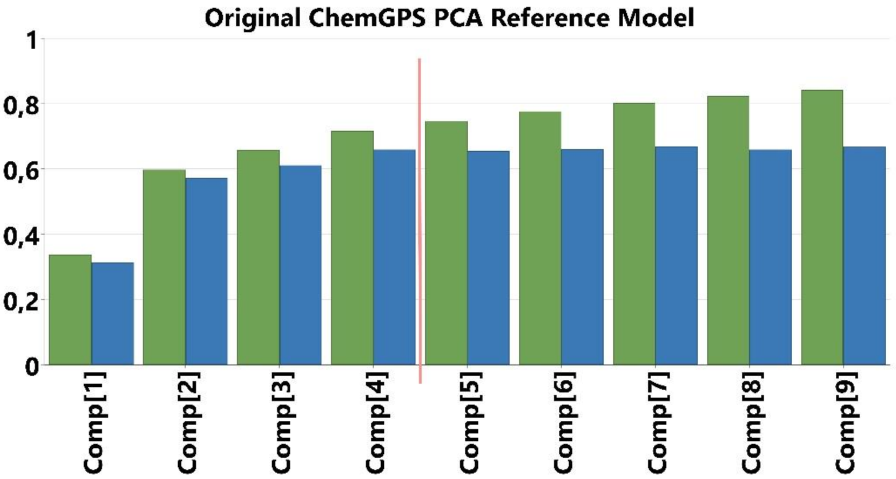

Figure S2: a) Overview of explained variance (R2) for the MOCA model M2. Explained variance R2 is separated into joint components (green) and unique components (blue). b) Evolution of explained variance (R2, green) and predicted variance (Q2, blue) per component of the PCA model in the original ChemGPS application. The red line

shows the “cut”, after 4 principal components, as the predictive power reaches a plateau and does not increase appreciably using five or more principal components.

Once extracted and combined as a new dataset SIMCA´s correlation matrix tool was used to compare all components on a pair-wise basis (**Figure S3**). The correlation matrix tool is intensity color coded in 11 steps. On the marked top right-hand part it is immediately clear that the first score vectors have much in common.

	M2.tj[1]	M2.tj[2]	M2.tj[3]	M2.tj[4]	M2.tj[5]	M2.tj[6]	M2.tj[7]	M2.tj[8]	M2.tj[9]	M2.tj[10]	M2.tj[11]	M2.tj[12]	M2.tj[13]	PCA t[1]	PCA t[2]	PCA t[3]	PCA t[4]
M2.tj[1]	1,00	0,00	0,00	0,02	-0,01	0,00	-0,01	0,00	0,00	0,00	0,01	0,01	0,00	0,95	-0,29	0,03	0,00
M2.tj[2]		1,00	0,00	0,04	0,01	0,00	0,00	0,00	0,00	0,00	0,00	0,00	0,00	-0,10	-0,45	-0,58	0,09
M2.tj[3]			1,00	-0,01	-0,01	-0,01	-0,02	0,00	0,00	0,00	-0,01	0,00	-0,01	0,24	0,72	-0,24	0,16
M2.tj[4]				1,00	0,00	0,01	-0,01	0,02	-0,01	0,00	-0,01	0,00	0,01	0,00	-0,18	-0,12	-0,03
M2.tj[5]					1,00	0,00	-0,02	0,01	0,03	0,02	0,00	0,01	0,00	-0,08	-0,17	-0,43	0,25
M2.tj[6]						1,00	0,02	0,00	-0,01	0,02	0,01	0,00	-0,01	-0,08	-0,24	0,53	0,13
M2.tj[7]							1,00	0,00	-0,01	0,05	0,01	-0,02	-0,02	-0,08	-0,18	0,24	0,43
M2.tj[8]								1,00	-0,02	0,00	-0,02	-0,02	0,01	-0,02	-0,03	-0,04	0,41
M2.tj[9]									1,00	0,04	0,03	0,02	0,01	0,04	0,09	-0,08	-0,10
M2.tj[10]										1,00	-0,03	0,01	0,01	0,01	0,00	0,01	0,20
M2.tj[11]											1,00	0,00	0,05	0,02	0,03	-0,08	-0,08
M2.tj[12]												1,00	0,04	-0,02	-0,06	0,03	0,02
M2.tj[13]													1,00	-0,02	-0,05	0,02	-0,24
PCA t[1]														1,00	0,00	0,00	0,00
PCA t[2]															1,00	0,00	0,00
PCA t[3]																1,00	-0,01
PCA t[4]																	1,00

Figure S3: Correlation matrix showing pair-wise correlations between the 13 joint components of the MOCA model M2 and the first 4 score vectors of the PCA model used in the original ChemGPS application.

An inspection of the correlation matrix reveals that, qualitatively, the first joint components resemble the ones of the original ChemGPS PCA model. For example, the scores of the first joint component of M2 and the first principal component of ChemGPS have a correlation coefficient exceeding 0.95 (**Figure S3**). **Figure S4** provides a scatter plot of the first joint component of M2 and the first principal component of the ChemGPS model.

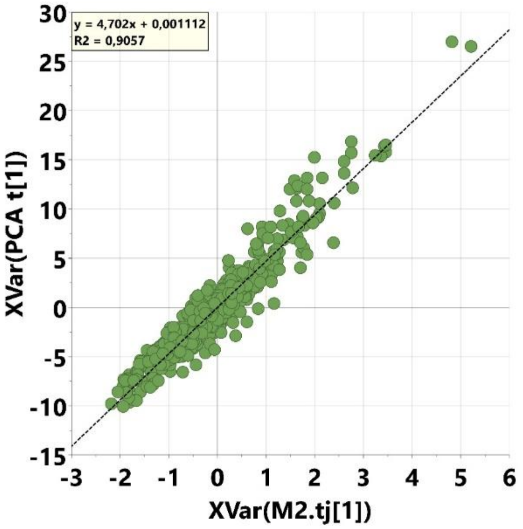

Figure S4: Scatter plot showing the correlation between the first PCA score vector (y-axis) and the first joint MOCA component (x-axis). The R2 of the correlation line is 0.9057, which is the square of the value 0.9516 found in the correlation matrix.

For the second and third joint components of M2 the second and third PCs of the ChemGPS model show the strongest correlations; however, there is a "smearing out" effect meaning that the two M2 joint components collectively correlate well with the two ChemGPS components.

Altogether, it is the first 8 joint components of the MOCA model that correlate with the 4 principal components of the ChemGPS original model. This higher number of correlating MOCA components is not entirely surprising as the MOCA model covers a much larger number of variables than the PCA model, i.e., there are more "latent variables" or "latent properties" of molecules encoded in the molecular descriptors underpinning the MOCA model.

In conclusion, the strong correlation and adherence to the structure of the ChemGPS model suggests the MOCA model M2 is well founded and provides faithful results.

3. Block wise elimination in MOCA model M2 on the ChemGPS data set

To test whether whole packages could be removed from a model without losing too much information can be achieved by block-wise elimination of each single block. We tested this approach for MOCA model M2 on the ChemGPS data set and looked at the explained variance R2 (joint + unique) of the remaining blocks (**Table S1**).

Table S1: Changes of explained variance R2 (join + unique) upon removal of one block from model M2

	M2	M2 without RDKit	M2 without CDK	M2 without cddd	M2 without QM	M2 without Dragon	M2 without PaDEL
RDKit	0.676	-	0.636	0.726	0.648	0.706	0.698
CDK	0.844	0.826	-	0.783	0.810	0.924	0.887
cddd	0.527	0.556	0.558	-	0.521	0.576	0.555
QM	0.633	0.587	0.640	0.855	-	0.718	0.611
Dragon	0.739	0.752	0.748	0.749	0.748	-	0.742
PaDEL	0.738	0.767	0.760	0.761	0.749	0.758	-
average abs. change	0	0.027	0.022	0.073	0.018	0.053	0.024

Elimination of the cddd and Dragon blocks changes the model structure significantly, which is also reflected in the R2 values of the remaining blocks. On the other hand, elimination of the QM block does not alter the remaining R2 values much but removes a number of unique descriptors.

Overall, this approach has a couple of difficulties and disadvantages:

- It is time consuming to build a new model for each removed block and analyze the results.
- Removal of a block can alter the model structure such that comparison to the original model becomes difficult, e.g. due to changes in the number or order of components.
- Removal of unique information from the eliminated block is not reflected in this approach.
- The fact that the R2 values were not altered significantly does not necessarily exclude qualitative shifts in the underlying components.

4. Correlation of joint components in model M3 on the Pesticides set

For each component SIMCA calculates the correlation (Pearson`s R2) of each block with all other blocks from all other components. This results in a matrix of size $((\#tj + \#tu) * \#blocks)^2$, which is called score correlation matrix. The full score correlation matrix can be obtained within SIMCA but is too large to be displayed here in detail. Therefore, we created a summarized version (**Figure S5**), in which the values for each joint component have been aggregated: minimum value for diagonal elements and maximum value for off-diagonal elements. From the off-diagonal elements it can be seen that the different joint components are not correlated with each other (maximum off-diagonal value between 0.00 and 0.30 for tj[1] and tj[6]), while the blocks are highly correlated within each component (minimum diagonal value between 0.44 for tj[8] and 0.91). Overall, this pattern of high correlation values for the diagonal elements and low values for off-diagonal elements indicate a sensible construction of the MOCA components. Relaxing the strictness of the MOCA model would likely lead to fewer MOCA components, where components with high correlation would be combined into one joint component. Depending on the user´s questions this can be the more appropriate choice.

For the unique components (not shown) the maximum correlation coefficient is 0.5.

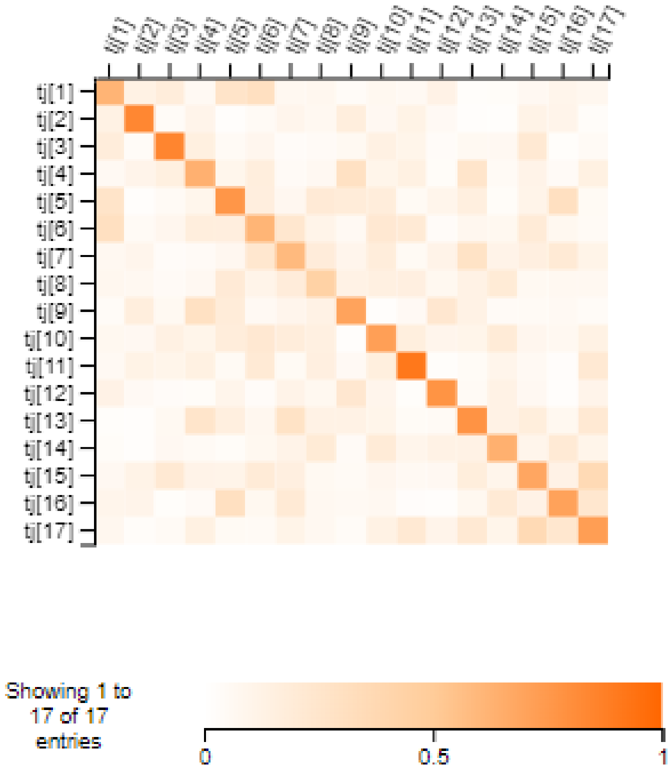

Figure S5: Grouped score-correlation matrix for model M3 on the Pesticides data set. For each component combination the minimum (diagonal elements) or maximum (off-diagonal) value from each block combination is shown.

The correlation coefficients for each individual block of joint components 1, 8 and 15 are given in **Table S2**.

Table S2: Model M3 score correlation matrix for joint components tj[1], tj[8], and tj[15]

	tj[1](bio)	tj[1](RDKit)	tj[1](cddd)	tj[1](QM)	tj[1](Dragon)	tj[1](PaDEL)
tj[1](bio)	1,00	0,62	0,67	0,62	0,71	0,68
tj[1](RDKit)	0,62	1,00	0,99	0,99	0,98	0,98
tj[1](cddd)	0,67	0,99	1,00	0,98	0,99	0,99
tj[1](QM)	0,62	0,99	0,98	1,00	0,98	0,98
tj[1](Dragon)	0,71	0,98	0,99	0,98	1,00	0,99
tj[1](PaDEL)	0,68	0,98	0,99	0,98	0,99	1,00

	tj[8](bio)	tj[8](RDKit)	tj[8](cddd)	tj[8](QM)	tj[8](Dragon)	tj[8](PaDEL)
tj[8](bio)	1,00	0,44	0,76		0,74	0,73
tj[8](RDKit)	0,44	1,00	0,62		0,62	0,53
tj[8](cddd)	0,76	0,62	1,00		0,83	0,81
tj[8](QM)						
tj[8](Dragon)	0,74	0,62	0,83		1,00	0,90
tj[8](PaDEL)	0,73	0,53	0,81		0,90	1,00

	tj[15](bio)	tj[15](RDKit)	tj[15](cddd)	tj[15](QM)	tj[15](Dragon)	tj[15](PaDEL)
tj[15](bio)	1,00		0,72			0,68
tj[15](RDKit)						
tj[15](cddd)	0,72		1,00			0,71
tj[15](QM)						
tj[15](Dragon)						
tj[15](PaDEL)	0,68		0,71			1,00

5. Systematic comparison of PLS models for individual blocks and ecotox endpoints

We constructed PLS models for all seven ecotoxicological endpoints and descriptor blocks individually. No further refinement of the models was attempted. The results are summarized in **Table S3**. The cddd block achieved the best scores for explained variance in most of the cases.

Table S3: PLS model comparison for each individual ecotox endpoint and descriptor block

		RDKit		CDK		cddd		QM		Dragon		PaDEL	
	n	R2	Q2	R2	Q2	R2	Q2	R2	Q2	R2	Q2	R2	Q2
algae	250	0.16	0.13	0.16	0.14	**0.27**	0.18	0.24	**0.20**	0.17	0.14	0.17	0.14
BCF	194	0.43	0.34	0.45	0.39	**0.65**	**0.39**	0.34	0.37	0.55	0.29	0.44	0.23
Birds	144	0.29	0.11	0*	0*	**0.72**	**0.26**	0*	0*	0.48	0.20	0.37	0.19
Daphnid	270	0.50	0.39	0.49	0.41	0.67	**0.53**	0.43	0.37	**0.74**	0.48	0.62	0.44
Aquatic plants	144	0.49	0.32	0.32	0.21	**0.60**	0.29	0.23	0.19	0.56	**0.37**	0.51	0.28
Fish acute	269	0.57	0.47	0.54	0.49	0.71	**0.56**	0.43	0.40	**0.81**	0.52	0.72	0.50
Rat acute	191	0.15	0.31	0.16	0.15	**0.63**	**0.34**	0.11	0.06	0.54	0.30	0.56	0.30
all targets	300	0.39	0.27	0.34	0.28	0.51	**0.35**	0.24	0.22	0.34	0.25	**0.53**	0.27
fish/daph	290	0.53	0.45	0.52	0.46	0.75	**0.55**	0.51	0.38	**0.82**	0.51	0.66	0.48

* no component was included in these models due to too little explained variance.
